# PCAF-mediated acetylation of Lin28B increases let-7 biogenesis in lung adenocarcinoma H1299 cells

**DOI:** 10.1186/s12885-017-3959-0

**Published:** 2018-01-04

**Authors:** Ting-ting Qu, Fei Chen, Jing Wang, Yan-jun Zhang, Mo-bin Cheng, Wen-zheng Sun, Yu-fei Shen, Ye Zhang

**Affiliations:** State Key Laboratory of Medical Molecular Biology, Department of Biochemistry and Molecular Biology, Institute of Basic Medical Sciences, Chinese Academy of Medical Sciences & School of Basic Medicine, Peking Union Medical College, 5 Dongdan Santiao, Beijing, 100005 China

**Keywords:** Lin28B, PCAF, let-7, Acetylation

## Abstract

**Background:**

Lin28B and its paralog Lin28A are small RNA binding proteins that have similar inhibitory effects, although they target separate steps in the maturation of let-7 miRNAs in mammalian cells. Because Lin28B participates in the promotion and development of tumors mostly by blocking the let-7 tumor suppressor family members, we sought to explore the associated mechanisms to gain insights into how Lin28B might be decreased in human cancer cells to increase let-7 levels and reverse malignancy.

**Results:**

We demonstrated that the histone acetyltransferase PCAF, via its cold shock domain, directly interacts with and subsequently acetylates Lin28B in lung adenocarcinoma-derived H1299 cells. RT-qPCR assays showed that both let-7a-1 and let-7g were increased in PCAF-transfected H1299 cells. Lin28B is acetylated by ectopic PCAF and translocates from the nucleus to the cytoplasm in H1299 cells.

**Conclusions:**

The effects of acetylated Lin28B on let-7a-1 and let-7g are similar to that of stable knockdown of Lin28B in H1299 cells. The new role of PCAF in mediating Lin28B acetylation and the specific release of its target microRNAs in H1299 cells may shed light on the potential application of let-7 in the clinical treatment of lung cancer patients.

## Background

Lin28 and the microRNA let-7 were first discovered in *C. elegans* as heterochronic genes that regulate developmental timing [1–3]. In eukaryotes including worms and mammals, Lin28 blocks let-7 expression, whereas let-7 negatively regulates Lin28 expression by binding to the 3’UTR of Lin28 mRNA, thereby establishing a double negative feedback loop. The Lin28/let-7 axis plays a pivotal role in stem cell biology and the development and control of glucose metabolism, as well as in human diseases [4, 5]. In mammals, there are two Lin28 paralogs: Lin28A and Lin28B. Although it is structurally similar to Lin28A, Lin28B contains a cold shock domain (CSD) and a retroviral-type CCHC zinc finger (ZF) motif. Lin28B has a coding extended C terminus that contains a nuclear localization signal (NLS) in addition to a nucleolus localization signal (NoLS) between the CSD and ZF domains, both of which participate in the subcellular localization of Lin28B in human cells [6–10]. The expression of Lin28A in the cytoplasm blocks let-7 processing by Dicer and uridylation of pre-let-7 by TUTase [11], whereas Lin28B primarily accumulates in the nucleus, where it binds pri-let-7 miRNAs and blocks the activity of the microprocessor complex [5, 8, 11]. However, the subcellular localization of Lin28B is controversial [4].

Lin28B was first cloned and identified as an over-expressed factor in hepatocellular carcinoma cells [6]. Lin28B is currently known to be involved in the promotion and development of tumors, thus indicating that it may be a potential target in human cancer therapy [7, 12–15]. A high Lin28A or Lin28B and low let-7 expression pattern is found in approximately 15% of human cancers [16]. The expression of Lin28B in cancer cells can be activated by transcription factors and epigenetic modifiers, such as Myc, NF-κB and Sirt6 [17–20]; however, much of the underlying mechanism remains unclear.

Acetylation is an important modification pattern that has been widely investigated in recent years. Protein acetylation is known to participate in regulating multiple cellular processes in normal and cancer cells [21–23]. As a bona fide cancer-related protein, Lin28B is subject to polyubiquitination that leads to the enhancement of let-7 biogenesis [24, 25]. However, whether the acetylation of Lin28B affects the let-7 biogenesis involved in tumorigenesis is not yet fully understood.

In this study, we found that knockdown of Lin28B in the human lung adenocarcinoma cell line H1299 abrogated the inhibition of let-7 miRNA. The histone acetyltransferase PCAF was found to directly interact with Lin28B via its CSD, and this interaction facilitated Lin28B acetylation by the HAT domain of PCAF. Most importantly, we demonstrated that the PCAF-mediated acetylation of Lin28B might de-repress the processing of let-7a-1 and let-7g, and these findings shed light on the potential application of acetylated Lin28B for future cancer therapy.

## Methods

### Cell culture

HEK293T, HCT116, MCF7, HeLa, HepG2, and H1299 cells were cultured in Dulbecco’s modified Eagle’s medium (DMEM) supplemented with 10% fetal bovine serum (FBS, HyClone) at 37 °C in 5% CO_2_ atmosphere. The HEK293T cells, MCF7, and H1299 cells were stored in our Lab. The HeLa (Cat. #3111C0001CCC000011) and HepG2 (Cat. #3111C0001CCC000035) cell lines were purchased from Chinese National Infrastructure of Cell Line Resource (Beijing, China). HCT116 cell line was a gift from Dr. Depei Liu (Institute of Basic Medical Sciences, Chinese Academy of Medical Sciences, Cat.# 3111C0001CCC000158). Before the experiments, the two cell lines were authenticated on cell micrograph compared to the cell lines on ATCC. HEK293T cells showed 90% transfect efficiency with GFP-tag plasmid. H1299 cells showed the lack of p53 protein expression by western blot assay. Mycoplasma contamination was detected by the EZ-PCR Mycoplasma Test Kit (Cat. #20–700-20), a PCR-based mycoplasma test kit, in cell cultures before the experiment. The kit includes a unique reaction mix that contains all the ingredients required for PCR: nucleotides, primers, Taq Polymerase and magnesium. After performing agarose gel electrophoresis, positive samples will yield a 270 bp fragment, but HEK293T and H1299 cell lines not.

We established two stably transfected clones of H1299 cells, in which Lin28B was knocked down by co-transduction of the cells with lentivirus encoding each of the shRNA specific for Lin28B, designated shLin28B-1, shLin28B-2, and shLuc was as a negative control. The shLin28B-1, shLin28B-2, or shLuc were cloned into the pLKO.1 vector. The shRNA sequences were as follows: shLin28B-1: GCAGGCATAATAAGCAAGTTA; shLin28B-2: GCCTTGAGTCAATACGGGTAA; shLuc: CGCTGAGTACTTCGAAATGTC.

### Antibodies

Antibodies against β-actin (sc-47778), GAPDH (sc-166545), Myc (sc-789), Lamin B (sc-6216) and PCAF (sc-13,124) were purchased from Santa Cruz Biotechnology (Santa Cruz, CA). Antibodies to FLAG (F3165) and M2 (F2426) were purchased from Sigma (St. Louis, MO). Antibodies to Lin28B (4196) and acetyl lysine (9441) were purchased from Cell Signaling Technology (Beverly, MA).

### Plasmids, constructs, and transfection

The FLAG-tagged and GFP-tagged Lin28B eukaryotic expression plasmids and GST-tagged Lin28B prokaryotic expression plasmids were constructed by cloning Lin28B into the pcDNA6-FLAG, pEGFP-C1-GFP and pGEX-4 T-1 vectors, respectively, using a PCR product from a cDNA library derived from HEK293T cells. pCX-FLAG-PCAF, pGEX-PCAF-HAT and pCX-FLAG-PCAF-∆HAT were as previously described [16]. Truncated fragments of Lin28B were cloned using the PCR product of full-length GFP-tagged Lin28B. FLAG-Lin28B-∆CSD was constructed by deleting the CSD. Myc-tagged PCAF was cloned from FLAG-tagged PCAF into the pCMV-3tag7-Myc vector. The eukaryotic expression plasmid of PCAF containing the Cys574Ser mutation was generated by site-directed mutagenesis of Myc-PCAF. The cells were transfected with Vigofect reagent (Vigorous Biotech, Beijing, China) according to the manufacturer’s instructions. Assays were performed 3 times each in triplicate, and all results are shown as the mean ± SD.

### Immunofluorescence

Cells were cultured on glass cover slips and fixed in 4% paraformaldehyde at room temperature for 10 min, washed three times with PBS for 15 min, permeabilized with 0.25% Triton X-100 in PBS for 15 min at room temperature, washed three times as described above and blocked with 1% BSA blocking solution at 37 °C for 1 h. The cells were incubated with primary anti-Lin28B antibody and anti-PCAF antibody at 1:50 dilution, anti-Myc antibody at 1:100 dilution and anti-FLAG antibody at 1:500 dilution at 4 °C overnight and washed three times with PBS. After washing, the cells were incubated with FITC- or TRITC-conjugated secondary antibodies at a dilution of 1:200 at 37 °C for 1 h and then washed three times with PBS. The coverslips were stained with DAPI, mounted and examined with a laser scanning confocal microscope.

### Co-immunoprecipitation (co-IP) and immunoblot analyses

Transiently transfected HEK293T cells were homogenized in lysis buffer (50 mM HEPES pH 7.5, 150 mM NaCl, 2 mM EDTA, 2 mM EGTA, 1% Triton X-100, 50 mM NaF, 5 mM Sodiun Pyrophosphate, 50 mM Sodium β-glycerophosphate, 1 mM NaVO_3_, 1 mM DTT, 1 mM PMSF, 10 μg/ml leupeptin and 10 μg/ml aprotinin). Cell lysates were rotated at 4 °C for 30 min and centrifuged at 12,000 rpm for 15 min to remove insoluble material and incubated with anti-GFP beads or anti-FLAG M2 beads at 4 °C overnight. The collected beads were then washed three times and boiled in SDS gel-loading buffer for western blot analysis.

### GST pull-down assay

The GST-tagged Lin28B fusion protein was expressed in *Escherichia coli* and purified using anti-GST beads. GST or GST-tagged Lin28B beads were individually incubated with cell lysates at 4 °C overnight. Finally, the beads were washed three times, and the bound proteins were analyzed by western blotting.

### Quantitative real-time RT-PCR assays (RT-qPCR)

RT-qPCR assays were carried out as described previously [26, 27]. Specific primers for Lin28B (forward: AGCCCCTTGGATATTCCAGTC and reverse: AATGTGAATTCCACTGGTTCTCCT). The relative expression of let-7a-1 and let-7g was normalized to that of U6 snRNA using the comparative CT method according to the manufacturer’s instructions (Bio-Rad CFX Connect Real-Time System). The primer sequences used for let-7 are listed below (F: forward; R: reverse; RT: reverse transcription). Mature let-7a-1 (RT: GTCGTATCCAGTGCAGGGTCCGAGGTATTCGCACTGGATACGACAACTAT, F: GCCGCTGAGGTAGTAGGTTGTA and R: GTGCAGGGTCCGAGGT), mature let-7g (RT: GTCGTATCCAGTGCAGGGTCCGAGGTATTCGCACTGGATACGACTTGACA, F: GCCGCTGAGGTAGTAGTTTGT and R: GTGCAGGGTCCGAGGT), mature mir-16-1 (RT: GTCGTATCCAGTGCAGGGTCCGAGGTATTCGCACTGGATACGACCGCCAA.

F: GCCGCTAGCAGCACGTAAATAT and R: GTGCAGGGTCCGAGGT) and U6 (RT: AAAATATGGAACGCTTCACGAATTTG, F: CTCGCTTCGGCAGCACA and R AACGCTTCACGAATTTGCGT) were used. The experiments were repeated at least three times with statistical analysis for individual experimental groups. All values were expressed as the mean ± SD.

### In vitro acetyltransferase assay

In vitro acetyltransferase assays were performed as previously described and detected by using western blotting with anti-acetyl lysine antibody [26]. Briefly, recombinant GST-Lin28B and GST-PCAF-HAT2 proteins were expressed in *E. coli* BL21 and purified using glutathione beads (Amersham Biosciences). For in vitro acetyltransferase assays, 1 μg of GST-PCAF-HAT2 and 5 μg of GST or GST-Lin28B were incubated in 25 μl of acetyltransferase assay buffer (50 mM Tris, pH 8, 10% glycerol, 10 mM butyric acid, 0.1 mM EDTA, 1 mM DTT, and 1 mM PMSF) with 20 μM acetyl CoA at 30 °C for 1 h. The reaction products were separated via 15% SDS-PAGE and analyzed by western blotting with anti-acetyl lysine antibody.

### Preparation of cell fractions

The cell fractions were prepared as previously described [28], and the prepared cell fractions were separated with 10% SDS-PAGE and detected by western blotting.

#### Statistical analysis

Statistical analysis was performed using two-tailed Student’s t-test. All data were shown as mean with standard deviations (SD). Probabilities of *P* > 0.05 were considered as no significant (#), *P* ≦ 0.05 as significant (*) and *P* ≦ 0.01 as highly significant (**).

## Results

### The effect of Lin28B knockdown on let-7 expression in H1299 cells

**Fig. 1:**
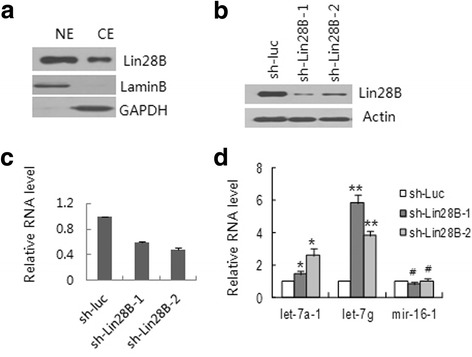
The effect of Lin28B knockdown on H1299 cells. **a** The nuclear and cytoplasmic distribution of Lin28B in H1299 cells. Nuclear (NE) and cytoplasmic (CE) extracts were immunoblotted with anti-Lin28B antibody. Lamin B: nuclear marker, GAPDH: cytoplasmic marker. **b** and **c)** The expression levels of Lin28B protein (**b**) and mRNA (**c**) in H1299 cells with Lin28B stably knocked down. Whole cell extracts (WCEs) from sh-Lin28B-1-, sh-Lin28B-2-, and sh-luc-transduced H1299 cells were subjected to gel electrophoresis and were blotted with antibodies, as shown on the right. β-actin: loading control (**b**); RT-qPCR. U6 snRNA was used as reference for mature miRNAs (**c**). **d** RT-qPCR analysis showing the levels of let-7a-1, let-7g and mir-16-1 in the control and Lin28B stable knockdown cells. Sh-luc: sh-RNA specific for luciferase as a negative control. In histograms, each bar represents a mean value ± S.D. from at least three independent experiments (#, *P* > 0.05; *p ≤ 0.05; **p ≤ 0.01)

Consistent with the literature [8], Lin28B is mainly found to be distributed in the nucleus (Fig. 1a), where it abrogates the expression of let-7 miRNA. To explore the functions of Lin28B in cancer cells, we first established two stably transfected clones of H1299 cells, in which Lin28B was knocked down by co-transduction of the cells with lentivirus encoding each of the shRNA specific for Lin28B, designated sh-Lin28B-1 and sh-Lin28–2. We found that the expression of the Lin28B protein (Fig. 1b) and its mRNA (Fig. 1c) was lower in the Lin28B-knockdown cells (Lin28B-K/D) than in cells mock transduced with shRNA against luciferase (sh-Luc). We then determined the expression of let-7 in H1299 cells and their Lin28B-K/D counterparts and found that knockdown of Lin28B enhanced the biogenesis of let-7 family members detected (let-7a-1, b, c, d, e, f, and g). The results of two representatives of let-7 miRNAs were shown in Fig. 1d, the let-7a-1 was increased and let-7g was drastically induced in Lin28B-K/D cells relative to their basal levels in the wild-type H1299 cells but mir-16-1 was not induced at all.

### PCAF is associated with Lin28B

**Fig. 2:**
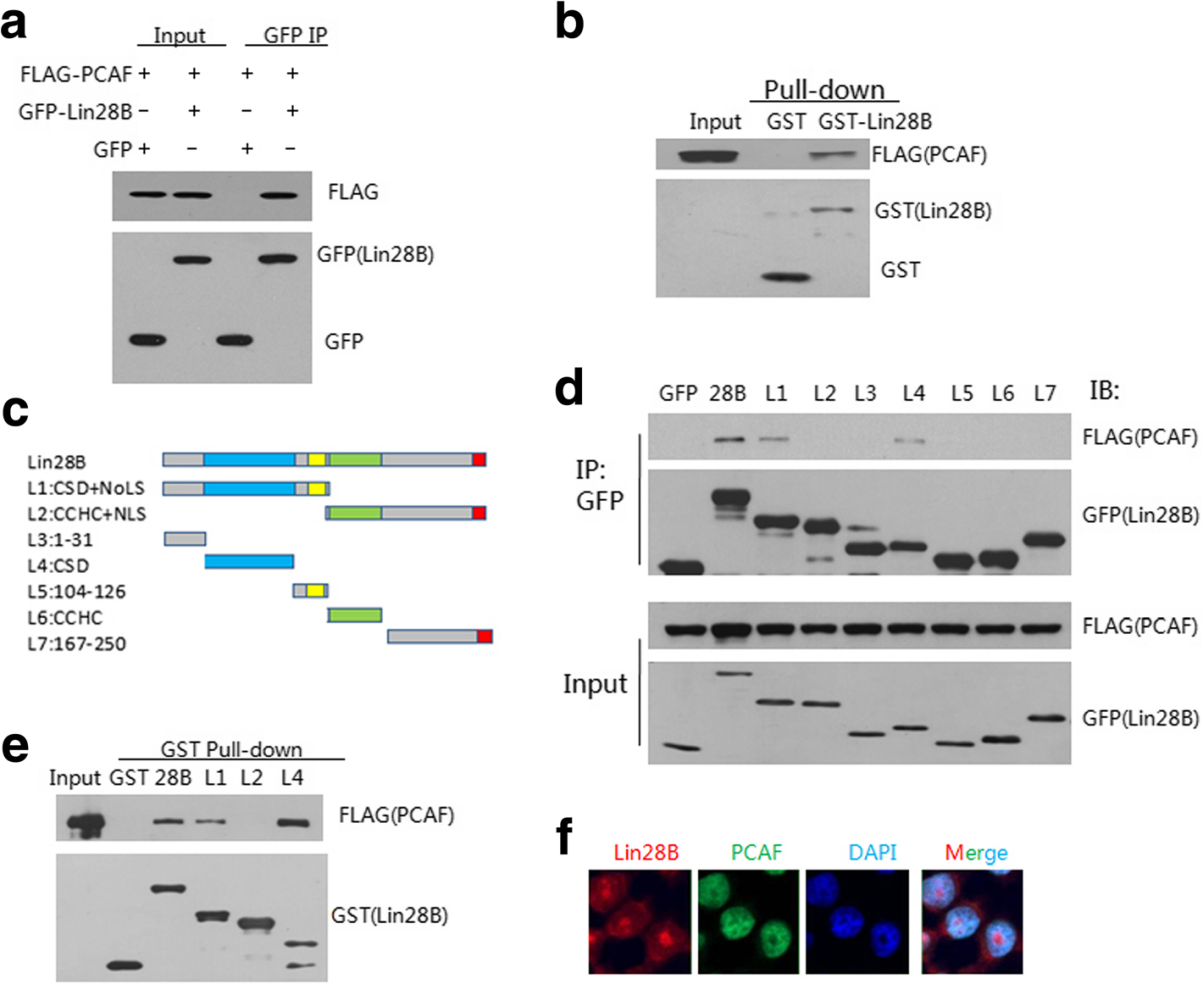
Lin28B interacts with PCAF via its cold shock domain. **a** Co-IP of the co-transfected PCAF-FLAG and Lin28B-GFP expression constructs in HEK293T cells. WCEs were IP-ed with anti-GFP antibody, and immunoblotted (IB) with the antibodies shown on the right. WCEs without immunoprecipitation are shown as Input. **b** GST-pulldown assay. Lin28B-GFP or GFP alone was individually incubated with WCEs of HEK293T cells ectopically expressing FLAG-PCAF, and the samples were subjected to gel electrophoresis and were blotted with antibodies shown on the right. **c** Schematic representation of the truncation fragments of Lin28B. Digits indicate the positions of the amino acids at the terminus of each fragment. **d** Domain-specific binding of Lin28B with PCAF. Each of the expression constructs of Lin28B and its individually truncated fragments was tagged with GFP (labeled as L1~L7) and individually co-transfected with PCAF-FLAG in HEK293T cells. WCEs of each transfected sample was IP-ed with anti-GFP antibody and then subjected to gel electrophoresis and was blotted with antibodies shown on the right. **c** GST-pull down assay identifying the interactions between specific Lin28B domains and PCAF in vitro. As shown in (**d**), each of the GST-tagged Lin28B and the related samples indicated at top was incubated with WCEs containing FLAG-tagged PCAF, and samples were then analyzed by western blotting with antibodies against GST and FLAG, as indicated. **f** Co-localization of Lin28B and PCAF in human cells. Immunofluorescence images showing endogenous Lin28B and PCAF in H1299 cells

In mammalian cells, PCAF acetylates numerous proteins with multiple roles in the normal growth and function of cells, and it is also associated with the occurrence of cancer [21–23]. Previously we reported that PCAF directly interacted with and acetylated Lin28A, the paralog of Lin28B [26]. To identify whether PCAF isassociated with Lin28Bwe assessed the interaction between PCAF and Lin28B by co-IP assay. Anti-GFP antibody was applied to co-immunoprecipitate GFP-tagged Lin28B with ectopic FLAG-PCAF in HEK293T cells (Fig. 2a). In addition, in vitro GST-pulldown assays indicated that FLAG-PCAF was pulled-down by GST-Lin28B in the HEK293T cell lysates (Fig. 2b). These results demonstrated an interaction between Lin28B and PCAF. To further identify the binding domain of Lin28B that interacts with PCAF, the coding region of the Lin28B gene was truncated; the representative protein fragments are shown in Fig. 2c. The co-IP assay revealed that the CSD of Lin28 (Fig. 2d, L1 & L4) was essential in mediating the interaction between Lin28B and PCAF. Similar results from in vitro GST-pulldown assays also indicated that the CSD of Lin28B mediated its binding with PCAF (Fig. 2e). Additionally, endogenous Lin28B- and PCAF-expressing H1299 cells were cultured on glass cover slips and treated with antibodies for immunofluorescence staining. Our results showed that Lin28B and PCAF co-localized in the nucleus of H1299 cells (Fig. 2f).

### PCAF acetylates Lin28B and involves in de-pression of let-7

**Fig. 3:**
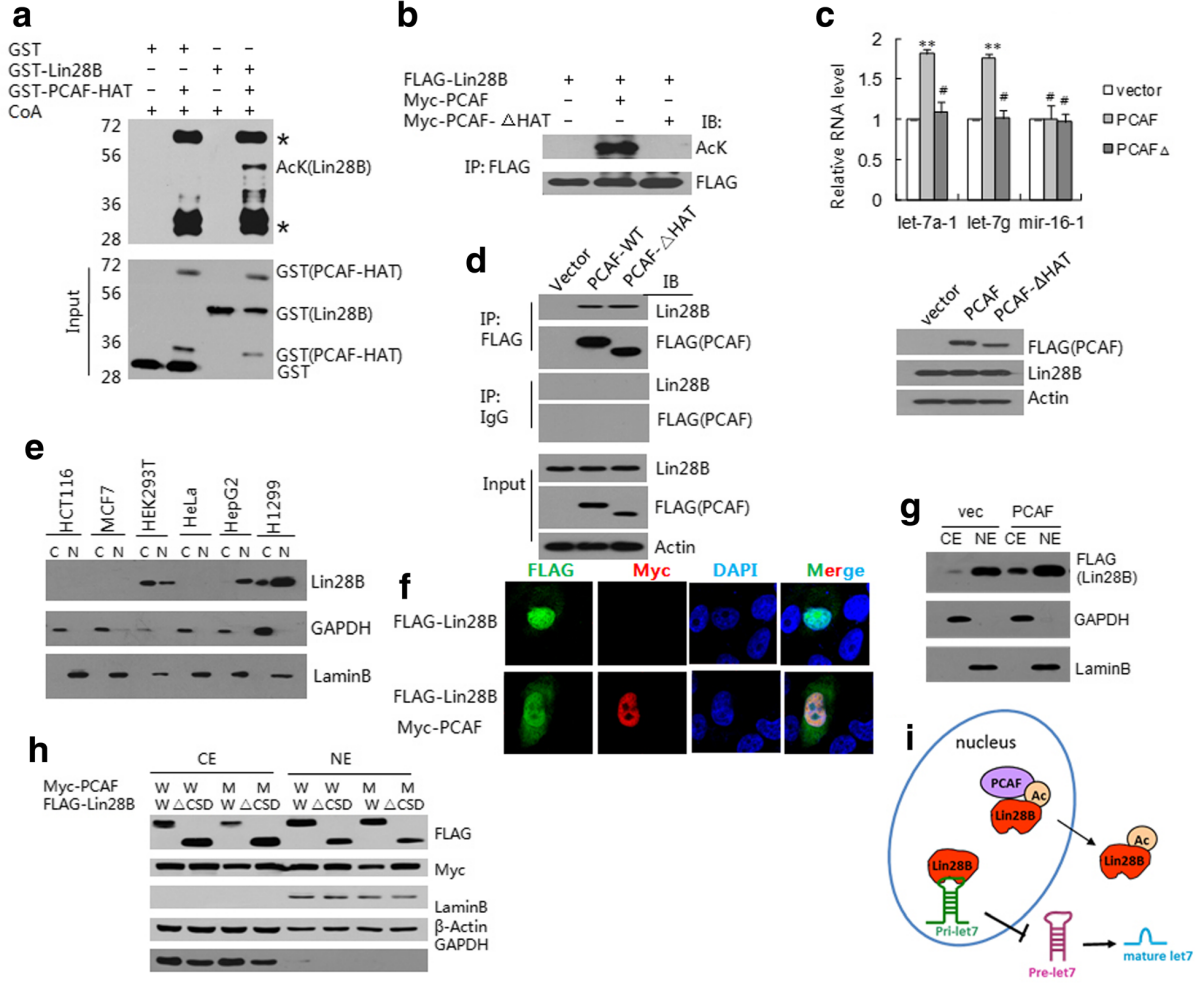
Acetylation of Lin28B by PCAF and its effect on the level of let-7. **a** In vitro acetyltransferase assay. GST-PCAF was incubated with GST-Lin28B in the presence of CoA, and blotting with antibodies against pan-acetyl lysine (AcK) and GST. * indicates autoacetylated PCAF-HAT and its derivatives. **b** Acetylation of Lin28B by PCAF is HAT domain-dependent. WCEs of HEK293T cells co-transfected with FLAG-Lin28B and either Myc-PCAF or Myc-PCAF-△HAT were IP-ed with anti-FLAG, then blotting with anti-AcK . and anti-FLAG. **c** Effect of PCAF on let-7. H1299 cells were transfected with FLAG-PCAF, FLAG-PCAF-△HAT or the vector. The expression levels of FLAG-PCAF and FLAG-PCAF-△HAT are shown at lower panel. RT-qPCR showing the levels of let-7a-1, let-7g and mir-16-1. Each bar represents the mean value ± S.D. from at least three independent experiments (#, *P* > 0.05; *p ≤ 0.05; **p ≤ 0.01, upper panel). **d** Interaction of PCAF with Lin28B is not dependent on the HAT domain. WCEs of FLAG-PCAF or PCAF-△HAT-transfected cells were IP-ed with anti-FLAG, then blotted with anti-FLAG and anti-Lin28B. IP with IgG served as a control. **e** Nuclear (NE) and cytoplasmic (CE) distribution of Lin28B. The extracts were blotted with anti-Lin28B. Lamin B: nuclear marker, GAPDH: cytoplasmic marker. **f** Localization of Lin28B and PCAF in H1299 cells. Immunofluorescence images showing ectopic Lin28B and PCAF in H1299 cells. **g** Effects of PCAF on the distribution of Lin28B. HeLa cells were co-transfected with FLAG-Lin28B and Myc-PCAF or vector (vec). CEs and NEs were immunoblotted with anti-FLAG. **h** The effects of PCAF on the distribution of Lin28B in H1299 cells. Cells were transfected with Myc-PCAF (wild-type, W; Cys574 mutant of PCAF, M) and FLAG-Lin28B (W, ΔCSD). CEs and NEs were immunoblotted with anti-FLAG and anti-Myc. **i** A schematic representation of the effect of PCAF-induced Lin28B acetylation on the synthesis of let-7

To explore whether Lin28B is acetylated by PCAF, an in vitro acetyltransferase assay was performed, as previously reported [26]. Using anti-acetyl lysine (AcK) antibody, we found that GST-Lin28B was acetylated only in the presence of the purified GST-fused HAT2-domain of PCAF (GST-PCAF-HAT), whereas PCAF was auto-acetylated (Fig. 3a). The expression constructs for FLAG-Lin28B were co-transfected into HEK293T cells with either Myc-PCAF or HAT-domain-deleted Myc-PCAF (Myc-PCAF-∆HAT). Ectopically expressed Lin28B was acetylated by Myc-tagged PCAF in the cells, whereas no acetylation of Lin28B was observed with the HAT-domain-deleted PCAF (Fig. 3b). To explore the functional role of PCAF compared with its HAT-domain-deleted counterpart in non-modified H1299 cells, RT-qPCR analysis was performed, and elevated expression levels of the let-7a-1 and let-7g miRNAs were found in the presence of PCAF but not the HAT-domain-deleted PCAF, whereas mir-16-1 was not induced by PCAF transfection (Fig. 3c). These results suggested that the acetylation of Lin28B by PCAF is a critical event that abrogates the inhibitory effect of native Lin28B on the biogenesis of let-7a-1 and let-7g miRNAs in H1299 cells.

We have previously reported that the protein stability of Lin28A, the paralog of Lin28B, decreases when it is acetylated by PCAF [26]; however, no apparent change was observed in the protein level of Lin28B in the cells transfected with PCAF (data not shown). Co-IP assays showed that PCAF-ΔHAT still interacted with Lin28B in cells (Fig. 3d).

To reveal the mechanism underlying the PCAF-mediated acetylation of Lin28B and its effect on the biogenesis of let-7, we first determined the expression of Lin28B in different cell types and detected it in three cell lines, H1299, HepG2, and HEK293T, but it was not detectable in other cell lines including HCT116, MCF7, and HeLa cells (Fig. 3e). However, unlike HEK293T cells, Lin28B was mainly distributed in the nucleus of H1299 cells (last group of cells in Fig. 3e). Furthermore, as compared with localization of Lin28A mostly in the cytoplasm but not in the nucleus [8, 26], Lin28B was mainly located in nucleus in these cells, and acetylation of Lin28B by ectopic PCAF enabled its translocation from the nucleus to the cytoplasm of H1299 cells, as shown by the results of immunofluorescence assays (Fig. 3f) and western blot assays on nuclear/cytoplasmic extracts (Fig. 3g). The distribution of endogenous Lin28B detected by anti-Lin28B is similar with that of ectopic Lin28B staining with anti-FLAG in H1299 cells (Fig. 2f vs Fig. 3f), which is confirmed with results of western blot for the fractions of nuclear/cytoplasmic (Fig. 1a vs the first two lines of Fig. 3g). To analyze the effects of PCAF activity on Lin28B, we used a Cys574 mutant of PCAF (M-PCAF) and Lin28B-ΔCSD and detected the cytoplasmic distribution of Lin28B in H1299 cells. The results showed that M-PCAF did not increase the cytoplasmic distribution of Lin28B and that the CSD-deleted Lin28B was mainly distributed in the cytoplasm and was not affected by M-PCAF (Fig. 3h). These findings indicated that the acetylation of Lin28B was induced by PCAF, which removed the inhibition on let-7 biogenesis in H1299 cells, as schematically shown in Fig. 3i.

## Discussion

In this report, we demonstrated that knockdown of Lin28B in H1299 cells elevated the level of let-7a-1/g, although this level of let-7a-1 was lower than that of let-7g in Lin28B-knockdown cells. The cell line H1299 was used as a model of lung adenocarcinoma and was derived from a lymph node metastasis of the lung. H1299 cells express high levels of Lin28B and low levels of let-7 [8, 16]. Lin28B, after acetylation by PCAF, might translocate from the nucleus to cytoplasm in H1299 cells and it may be further involved in the de-repression of let-7 biogenesis. These findings provide new insights into the potential application of acetylated Lin28B in mediating let-7 biogenesis, which may serve as a novel therapeutic approach for lung cancer.

In humans, there are twelve let-7 family members located at eight different chromosomal loci. Lin28A/B selectively represses the expression of let-7 miRNAs [11, 29–31]. It is well known that cytoplasmic Lin28A first binds to the conserved terminal loop of pre-let-7 and then recruits TUT4 for polyuridylation, thereby blocking Dicer cleavage [4, 5]. Although the precise mechanism underlying the Lin28B-mediated inhibition of let-7 remains controversial, it has been demonstrated that Lin28B expression and let-7 loss almost invariably correlate with poor prognosis [4]. Activation of Lin28B expression in cancer cells can be triggered by upstream transcriptional factors, such as c-Myc [18, 19] and NF-κB [20]. In addition, it has been reported that Merlin/NF2 is a key regulator of Lin28B localization and let-7 biogenesis in response to cell-cell contact [32]. Our data suggested that acetylation of Lin28B by PCAF may affect Lin28B localization and let-7 biogenesis.

Although we have previously reported that the protein level of Lin28A had decreases in PCAF-transfected cells [26], Lin28B was not substantially changed at the protein level (data not shown). Both Lin28A and Lin28B interact with PCAF via their CSD, and let-7 binds to the 3’UTR of both Lin28 paralogs, thereby repressing Lin28 translation in a negative feedback loop; however, TRIM71, a specific E3 ubiquitin ligase, negatively regulates Lin28B (but not Lin28A) protein levels by ubiquitinating its C-terminal unique region, which is absent in the Lin28A paralog [24, 25]. It might be interesting to study whether the acetylation of Lin28B by PCAF affects the poly-ubiquitination of Lin28B at the C-terminal region and may alter its stability.

## Conclusion

In summary, we demonstrated that Lin28B interacts directly with PCAF via its cold shock domain and is acetylated by PACF. The HAT domain of PCAF is indispensable for PCAF mediated acetylation of Lin28B and de-repression of let-7a-1 and let-7g. Lin28B, after acetylation by PCAF, might translocate from the nucleus to cytoplasm in H1299 cells and it may be further involved in the de-repression of let-7 biogenesis. The discovery of a new role of PCAF in mediating Lin28B acetylation and, particularly, in elevating the level of *microRNAs* in lung adenocarcinoma-derived H1299 cells may shed new light on the potential application of let-7a-1 and/or let-7g in the clinical treatment of lung cancer.

## Abbreviations

AcK: Acetyl lysine
Co-IP: Co-immunoprecipitation
CSD: Cold shock domain
DMEM: Dulbecco’s modified Eagle’s medium
Lin28B-K/D: Lin28B-knockdown cell lines
NLS: Nucleus localization signal
NoLS: Nucleolus localization signal
RT-qPCR: Quantitative real-time RT-PCR assays
ZF: CCHC zinc finger motif
